# Impact of sheath diameter of different sheath types on vascular complications and mortality in transfemoral TAVI approaches using the Proglide closure device

**DOI:** 10.1371/journal.pone.0183658

**Published:** 2017-08-24

**Authors:** Zisis Dimitriadis, Werner Scholtz, Stephan M. Ensminger, Cornelia Piper, Thomas Bitter, Marcus Wiemer, Marios Vlachojannis, Jochen Börgermann, Lothar Faber, Dieter Horstkotte, Jan Gummert, Smita Scholtz

**Affiliations:** 1 Clinic for Cardiology, Herz- und Diabeteszentrum NRW, Ruhr-Universität Bochum, Bad Oeynhausen, Germany; 2 Clinic for Thoracic and Cardiovascular Surgery, Herz- und Diabeteszentrum NRW, Ruhr-Universität Bochum, Bad Oeynhausen, Germany; 3 Department of Cardiology and Critical Care Medicine, Johannes-Wesling-Klinikum Minden, Ruhr-Universität Bochum, Minden, Germany; Klinikum Region Hannover GmbH, GERMANY

## Abstract

**Objective:**

Evaluation of the impact of the sheath diameter on vascular complications and mortality in transfemoral aortic valve implantation.

**Method:**

Between 2012 and 2014, 183 patients underwent the procedure using a sheath diameter of 18–24 F. This collective was divided into two groups: group 1, with a sheath diameter of 18F (G1, n = 94), consisted of patients with 18F Medtronic Sentrant and 18 F Direct Flow sheaths, and group 2 with a sheath diameter of 19–24 F (G2, n = 89) consisted of patients with Edwards expandable e-sheath and Solopath sheaths. Perclose-Proglide^®^ was used as a closure device in all patients.

**Results:**

G1 had significantly more female patients (64.9% vs. 46.1% in G2, p = 0.01) and the average BMI was lower (26 ± 4.5% vs. 27.4 ± 4.7%, p = 0.03). There was no significant difference in the incidence of major and minor vascular complications (G1: 12.8% vs. G2: 12.4%, p = 0.9). 30-day mortality was similar in both groups (G1: 6.4 ± 2.5% [95% CI: 0.88–0.98], G2: 3.7 ± 1.9% [95% CI: 0.92–0.99]. The Kaplan Meier analysis of survival revealed no significant differences either.

**Conclusion:**

The difference in sheath diameter had no effect on either incidence or severity of vascular complications. There was no impact on mortality either.

## Introduction

For decades, surgical aortic valve replacement has been the standard treatment for patients with severe aortic stenosis (AS). However, this procedure entails higher risks for patients > 80 years of age, especially when comorbidities are present, such as coronary artery disease, systolic heart failure, cerebrovascular and peripheral arterial diseases, chronic respiratory dysfunction or chronic renal disease, and others [1]. In 2002, the first transcatheter aortic valve implantation (TAVI) was performed providing a feasible alternative for high-risk patients [2,3]. Since then, around 100,000 successful TAVI procedures have been reported with outcomes comparable to conventional aortic valve surgery [4–9]. For TAVI, several alternative approaches have been developed, such as transapical, subclavian, direct aortic, or transcaval route. However, transfemoral (TF) TAVI is the least invasive and is most widely applied [10–12]. Initially, femoral access and closure required surgical cut-down with arteriotomy, which has the additional drawbacks of general or spinal anesthesia and longer duration of surgery and convalescence [13]. With the introduction of closure devices, such as Prostar XL and Perclose-Proglide^®^ closure devices (both from Abbott Vascular Inc., Santa Clara, CA, USA), risks of surgical cut down have been reduced significantly, especially after an adequate learning curve [14,15]. Nevertheless, TF-TAVI is associated with vascular complications coupled with a significant impact on morbidity and mortality rates [16–18]. Since TAVI is a relatively new technique, large-scale studies revealing the major factors contributing to minor and major vascular complications remain scarce. Although several aspects thereof have been addressed, such as patient selection criteria, technical advancements of the bio-prosthetic valve devices (either balloon-expandable e.g., Edward SAPIEN, or self-expandable e.g., Medtronic CoreValve), the issue requires further attention [9,19].

Because the sheath diameter has been identified as an independent predictive factor of vascular complications in endovascular aortic aneurysm repair [20,21], it is believed to have a similar impact in TAVI [22]. The aim of this study was to clarify the impact of the sheath diameter of different sheath types in TF-TAVI patients, using the Perclose-Proglide^®^ closure device, on the incidence of vascular complications (as defined by the VARC consensus) and mortality.

## Patients and methods

### Patients

Patients were considered for TAVI if they had severe symptomatic aortic valve stenosis and were classified to be at high risk for conventional surgery due to comorbidities, age, or the presence of a porcelain aorta. All patients underwent coronary angiography, and if significant coronary artery disease (CAD) was present, percutaneous coronary intervention was performed prior to TAVI. Multi-slice computed tomography (MSCT) was part of the screening process in all patients. The MSCT data set was analyzed using the dedicated software 3Mensio Structural Heart (3Mensio Structural Heart, Pie Medical Imaging, Maastricht, the Netherlands) in order to determine the type and size of the prosthetic valve as well as the type of access. The European System for Cardiac Operative Risk Evaluation (EuroSCORE) I, EuroSCORE II, and Society of Thoracic Surgeons (STS) scores were calculated as part of the evaluation. All cases were discussed with, and the indications confirmed by, the institutional heart team. The study was approved by the local ethics committee (Ethikkommission der Medizinischen Fakultät der Ruhr-Universität Bochum, Sitz Bad Oeynhausen; No 3/2016, dd. 24 February 2016). Written informed consent was obtained regarding use of data for scientific purposes.

Between 2012 and 2014 the TAVI-TF procedure was performed in 183 patients. The following valve types were implanted: Medtronic CoreValve^®^ (Medtronic, Minneapolis, MN, USA), Edwards SAPIEN Valve (Edwards Lifesciences, Irvine, CA, USA), and Direct Flow Valve (Direct Flow Medical Inc., Santa Rosa, CA, USA).

### TAVI procedure

All TAVI-TF procedures were performed in a hybrid operating room equipped with a Siemens Artis Zeego imaging system. The interventions were conducted under fluoroscopic guidance with patients under conscious sedation and local anesthesia. Balloon pre-dilation of the native aortic valve was performed under rapid pacing in the early stage of our TAVI program, but since 2012, Edwards and CoreValve devices have been implanted directly into the native valve without pre-dilation. Before the procedure, any oral anticoagulation therapy was stopped and the International Normalized Ratio (INR) had dropped below 2.0. Hemodynamic measurements were taken before and directly after valve implantation. A final angiogram with 30 ml of a contrast agent at a flow rate of 15 ml/s was performed to ascertain the final valve position and to make residual paravalvular leakage visible. Heparin was administered at the beginning to keep the activated clotting time (ACT) above 250 s throughout the procedure. Heparin was neutralized by protamine at the end of the procedure. After closure of the puncture site with Proglide^®^, we applied a pressure bandage for 4 hours.

All patients were administered a daily dose of 100 mg acetylsalicylic acid (ASA) if they were under no oral anticoagulation, and patients with Medtronic CoreValve and Direct Flow Medical implantation received 75 mg Clopidogrel/day in addition to ASA or oral anticoagulation for three months after the procedure.

### Closure devices

In all 183 patients, two Perclose-Proglide^®^ Suture-Mediated Closure System (Abbott Vascular, Redwood City, CA; USA) devices were used per case to close the puncture site of the TAVI sheath. The mechanism and design of the closure device has been described previously [23]. After closure of the puncture site, its effectiveness was verified angiographically by contrast-agent injection in the femoral artery from the contralateral side.

### Sheaths

The following sheaths were used for advancing the different valve types: 18 F Medtronic Sentrant Introducer sheath or 18 F Cook sheath for the Medtronic CoreValve; the 16/18/20 F expandable E-Sheaths for the SAPIEN XT and SAPIEN 3 Edwards Valves; the 18 F Direct Flow sheath for the Direct Flow Valve and, in a few cases, the 14 F expandable Solopath (SoloPath^™^-Introducer, Onset Medical Corporation, Irvine, CA, USA), which can be expanded up to 19 F. After expansion, 16F and 18F eSheaths become 22F, and the 20F sheath 26F.

The sheath to femoral artery ratio (SFAR) describes the ratio between the sheath’s outer diameter and the femoral artery minimal luminal diameter, and was calculated for the whole collective. The SFAR analysis was performed with the outer sheath diameter in the expanded status.

The collective was divided into two groups, group 1 with a sheath diameter of 18F (G1, n = 94), and group 2 with a sheath diameter of 19–24 F (G2, n = 89), depending on the final sheath diameter, meaning the final eSheath diameter after expansion. The 16 F eSheath has a 22F diameter after expansion, like the 18F eSheath. The patients treated with a 16F eSheath were also included in the G2 group.

### Post-procedure clinical evaluation

After the procedure, all patients were transferred to the intensive care unit. Echocardiography and clinical examinations were performed immediately thereafter. After removal of the pressure bandage from the groin, a further clinical examination with auscultation of the puncture site was performed followed by sonography. Clinical end points were assessed according to the updated Valve Academic Research Consortium Criteria [24].

### Follow-up

Patients were reevaluated at three and twelve months after the TAVI procedure in our out-patient department. Clinical and echocardiographic findings were recorded. In all cases, patients were followed every year up to the third year in a standardized telephone interview documenting their general health status and cardiovascular events.

### Statistical analysis

Descriptive statistics were used to evaluate demographic data. Categorical variables were presented as frequencies and percentages, and continuous variables as mean ± standard deviation (SD). Baseline data were checked for normal distribution using the Kolmogorov-Smirnov method. A two-tailed unpaired Student *t* test was used for comparison of continuous data between groups and a paired Student *t* test for intragroup comparison. Ordinal variables were compared with the Mann-Whitney *U* test and Wilcoxon signed-rank test. A chi-square test was used in order to investigate the relationship between two categorical variables.

Survival curves were calculated using Kaplan-Meier analysis and were compared among groups by means of a log-rank test. Cox proportional hazards models were used to investigate two or more factors for survival, to calculate hazard ratios (HZ), and to test for interactions. A univariate Cox regression analysis was performed to evaluate the impact on mortality. Parameters with p < 0.1 in the univariate Cox regression analysis were included in the multivariate Cox regression analysis.

After evaluation of multicollinearity, a hierarchical regression analysis was performed to evaluate the effect on the clinical end points as continuous variables. The following parameters were included: sex, age, body mass index (BMI), sheath diameter, anticoagulation, peripheral artery disease (PAD), vessel diameter, STS score, and EuroSCORE II.

A two-tailed p-value of < 0.05 was considered to indicate statistical significance. Commercially available software was used for analyses (SPSS 22.0, SPSS Inc., Chicago, Illinois, USA).

## Results

### Baseline characteristics and complications of the entire study population

Table 1 presents the baseline characteristics of the entire collective. The Medtronic Sheath was applied in 86 patients (47%), the eSheath in 88 patients (48%), the Direct Flow Sheath in 8 patients (4.4%) and the SoloPath in 1 patient (0.5%). Table 2 shows the complications observed in the entire study population in details. The average follow-up was 496 ± 281 days.

**Table 1:** Baseline characteristics of the entire collective. (BMI = body mass index, LV EF = left ventricular ejection fraction, AV = aortic valve, PAD = peripheral artery disease, COPD = chronic obstructive pulmonary disease, CAD = cardiac artery disease). Variables are expressed as mean ± standard deviation (median) or percentage (number).

Baseline Characteristics	n = 183
Female (n)	56.2% (103)
Age (years)	82.6 ± 5 (83)
BMI (%)	26 ± 4.6 (26.4)
LV EF (%)	53.5 ± 10.2 (60)
AV orifice (cm^2^)	0.7 ± 0.2 (0.8)
AV pressure (mmHg)	45.1 ± 17.1 (41.5)
Euroscore II (%)	6 ± 5.6 (4.4)
STS Score (%)	5.7 ± 4.3 (5)
Common femoral artery diameter (mm)	8.2 ± 1.9 (8)
Anticoagulation	31.7% (58)
PAD	8.7% (16)
COPD	14.8% (27)
Diabetes mellitus	23.5% (43)
Hypertension	65% (119)
CAD	54.6% (100)
Renal failure	38.2% (70)
SFAR	1.02 ± 0.19 (1.03)

**Table 2:** Vascular complications in the entire collective. The second part of the table summarizes the complications according to the VARC-II classification. Variables are expressed as mean ± standard deviation (median) or percentage (number).

Complications	
Stenosis	9.3% (17)
Occlusion	1.3% (2)
Dissection	6% (11)
Surgical treatment	1.3% (2)
Suture rupture	1.3% (2)
Bleeding	7.1% (13)
Endovascular stenting	5.5% (10)
Pseudoaneurysms	1.6% (3)
No VARC-II complication	87.4% (160)
Minor VARC-II complication	8.7% (16)
Major VARC-II complication	3.8% (7)
All-cause mortality during follow up	19.9% (36)

### Baseline characteristics and complications compared between the two study groups

G1 included 86 patients with the Medtronic sheath and 8 patients with the Direct Flow sheath, G2 1 patient with the SoloPath sheath (19 F after expansion), 9 patients with the 16 F eSheath (22 F after expansion), 38 with the 18 F eSheath (22 F after expansion), and 41 with the 20 F eSheath (26 F after expansion).

Only three aspects of the baseline data were significantly different between the two groups (Table 3). G1 had significantly more females, a higher BMI and a lower SFAR compared to G2. Furthermore, it is worth mentioning that in G1, the Medtronic sheath was used more often, while in G2, the eSheath was applied more often (91.5% vs. 98.9%, p < 0.001). The vascular complications were not significantly different between patients with small and large sheaths (Table 4). Further analysis also revealed no significant differences according the VARC-II classification. The multivariate analysis showed no significant impact of any of the analyzed parameters on the vascular complications (Table 5). Interestingly, even a SFAR ≤ 1.05 was not identified as a predictive factor for vascular complications in our study.

**Table 3:** Baseline characteristics compared between the 18 F and 19–24 F group. Variables are expressed as mean ± standard deviation (median) or percentage (number).

	Sheath 18 F (n = 94)	Sheath 19–24 F (n = 89)	P
Female (n)	64.9% (61)	46.1% (41)	0.01
Age (years)	82.7 ± 5.6 (83)	82.6 ± 4.3 (83)	0.9
BMI (%)	26 ± 4.5 (25.7)	27.4 ± 4.7 (26.9)	0.03
LV EF (%)	53.6 ± 10.3 (60)	53.5 ± 10.2 (60)	0.8
AV orifice (cm^2^)	0.72 ± 0.17 (0.7)	0.77 ± 0.17 (0.8)	0.07
AV Pressure (mmHg)	45.6 ± 17.9 (43)	44.8 ± 16.1 (41)	0.8
Euroscore II (%)	6.4 ± 5.9 (4.7)	5.7 ± 5.3 (4.1)	0.2
STS Score (%)	5.8 ± 4.8 (5.2)	5.8 ± 3.7 (4.9)	0.2
Common femoral artery diameter (mm)	7.9 ± 1.9 (8)	8.3 ± 1.8 (8)	0.09
Anticoagulation	33% (31)	29.1% (26)	0.6
PAD	6.4% (6)	9% (8)	0.4
COPD	14.9% (14)	14.6% (13)	0.6
Diabetes mellitus	24.5% (23)	22.5% (20)	0.6
Hypertension	64.9% (61)	62.9% (56)	0.6
CAD	46.8% (44)	60.7% (54)	0.1
Renal failure	39.6% (37)	34.8% (31)	0.3
SFAR	0.92 ± 0.13 (0.9)	1.1 ± 0.18 (1.1)	< 0.001

**Table 4:** Complications compared between each group. The first part of the table presents the individual complications, and the second part the complications is grouped according to the VARC-II classification. Variables are expressed as percentage (number).

	Sheath 18F (n = 94)	Sheath 19-24F (n = 89)	p
Stenosis	12.8% (12)	5.6% (5)	0.1
Occlusion	5.3% (0)	2.2% (2)	0.2
Dissection	5.3% (5)	6.7% (6)	0.7
Surgical treatment	0% (0)	2.2% (2)	0.2
Suture rupture	0% (0)	2.2% (2)	0.2
Bleeding	6.4% (6)	7.9% (7)	0.8
Endovascular stenting	4.3% (4)	6.7% (6)	0.5
Pseudoaneurysms	1.1% (1)	2.2% (2)	0.6
No VARC-II complication	87.2% (82)	87.6% (78)	0.9
Minor VARC-II complication	9.6% (9)	7.8% (7)	
Major VARC-II complication	3.2% (3)	4.5% (4)	

**Table 5:** Results of the multivariate analysis regarding the vascular complications.

	OR	95% CI	P
Sex	1	0.4–21	0.3
Age	1	0.8–1	0.3
BMI	1	0.8–1.1	0.3
Sheath type	1	0.3–3.2	0.5
Anticoagulation	1	0.2–7.2	0.8
PAD	1.2	0.3–4.7	0.4
Vessel diameter	0.3	0.1–66.7	1
EuroScore II	0.7	0.2–3.8	0.9
STS Score	2.3	0.2–9.2	0.8

### Mortality

**Fig 1:**
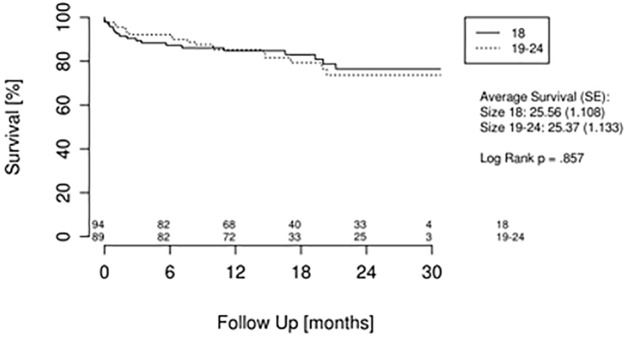
Kaplan-Meier mortality analysis in relation to sheath diameter.

In accordance with our data, 30-day mortality showed no significant difference between the groups with 6.4 ± 2.5% (95% CI: 0.88–0.98) in G1 and 3.7 ± 1.9% (95% CI: 0.92–0.99) in G2. The same was true for survival after analysis of the complete follow-up. The Kaplan Meier analysis is presented in Fig 1. After performing a univariate Cox analysis, the following parameters could be included in the multivariate Cox regression analysis: sheath type, vessel diameter, EuroScore II, STS Score, LV EF, and bleeding. The multivariate Cox regression analysis revealed the following significant parameters: vessel diameter (HZ: 5.1, 95% CI: 1.4–18, p = 0.01), STS Score (HZ: 3.3, 95% CI: 1.4–8.1, p = 0.005), and bleeding (HZ: 1.1, 95% CI: 1–1.1, p = 0.05).

## Discussion

### Vascular complications in TF-TAVI

After its introduction in 2002, TAVI presented a valuable alternative for previously inoperable AS patients, or those with high surgical risk. Application of a percutaneous procedure can reduce the need for general or spinal anesthesia, duration of surgery, risk of infections, post-operative patient immobilization and discomfort, and convalescence [13]. Despite many improvements to the technique, percutaneous TAVI is still associated with significant risks of minor and major vascular complications. This remains an important issue, as major vascular complications are an independent predictor of short-term mortality [9]. Reports on the incidence of TAVI-related vascular complications vary between 5 and 30% [14,18,25]. This can be explained by an initial lack of clear definitions and graduations of vascular complications as well as by continuous improvements of both surgical skills and device designs. In particular, the impact of the surgeon’s learning curve has been demonstrated repeatedly [14,20]. In Valve Academic Research Consortium (VARC), Leon et al. formulated clear endpoints for vascular complications, which allow for comparison and meta-analysis using independent studies [26].

According to the updated VARC II criteria, we found 12.6% vascular complications in all patients in our study, 3.8% of which were major complications. These numbers are comparable with those of recent reports [14,27–29]. The study emphasizes two important points. First, we analyzed vascular complications according to the final diameter and not the diameter at the beginning of the procedure prior to sheath expansion. Such an expansion dilates the puncture site and presses calcifications in the femoral and iliacal artery, which might lead to a dissection. This point was also taken into account in the SFAR analysis. Secondly, we analyzed vascular complications only in patients treated with the Proglide^®^ closure device. Between the two commonly used closure devices, Prostar^®^ and Proglide^®^, there are differences with respect to the vascular complications [30,31]. According to our knowledge this is the first study reporting on a detailed analysis of vascular complications depending on sheath diameter using only the Proglide^®^ closure device.

Nevertheless, it must be mentioned that four different types of sheath were applied in this study, with not only different diameters but also different characteristics and performance parameters, especially in terms of trackability and pushability. An important difference is the expandability of the eSheath. This feature is based on the special design of the sheath with a slit along its flexible part allowing for short-term expansion during advancement. This is an important difference because, due to the number of cases, the main comparison was made between the 18F Medtronic Sentrant and the Edwards expandable e-sheath. The differences in design and performance of the sheaths may also have an impact on vascular complications, and must therefore be taken into account.

The incidence of vascular complications in our TF-TAVI patients treated with the Perclose-Proglide^®^ closure device was 12.8% and 12.4% in G1 and G2 respectively, while major vascular complication occurred in 3.2% and 4.5% of patients in the respective groups. Griese et al. (2013) and Barbash et al. (2015) reported similar complication rates when using the Perclose-Proglide^®^ closure device for TAVI procedures [23,31]. These low numbers indicate that the use of this device for the closure of the punctured femoral artery is effective and safe, as confirmed by others [23,32]. Several predictors of vascular complications after TF-TAVI have been previously identified, such as center and surgeon experience, femoral artery calcification, minimal artery diameter, and SFAR ≥ 1.05 [33,34]. Our results show no significant difference in the incidence of vascular complications between the two groups, indicating that sheath diameter does not impact on vascular adverse events. This same conclusion was reached by Hayashida et al. (2012) [14], Greason et al. (2013) [35] and Borz et al. (2014) [28].

Nevertheless, vascular complications in transfemoral TAVI procedures are a very complex issue and different parameters like age, patient selection, peripheral artery diseases, access-site calcifications, operator experience, valve type, and improvements in devices and techniques have to be considered as potentially influencing factors on vascular complication rate and follow-up procedures. This is the case even though these factors do not return statistically significant baseline differences or show significance in the regression analysis in our study. Sex, BMI and SFAR, which show a statistically significant baseline difference between our collectives, could also impact on the results, although neither the multivariate regression analysis with respect to vascular complications nor the multivariate Cox regression analysis revealed a significant impact of these parameters.

Hayashida et al., who defined the parameter SFAR for the first time in 2011, did observe a correlation between the SFAR and the incidence of vascular complications after TF-TAVI, and that SFAR ≥ 1.05 was predictive of a higher risk of vascular complications [10]. Recently however, Krishnaswamy et al (2014) applied computed tomography to analyze risk factors for vascular complications in the setting of TF-TAVI, and found the cut-off value of the risk impact of SFAR to be 1.45 instead [36]. Interestingly, several other studies do report a significant impact of sheath diameter in addition to observations that SFAR is a predictive factor for vascular complications [29,30]. As such, Barbanti et al (2013) found a complication rate of 0.5% when low diameter sheaths (14-18F) were used compared to 10.5% when the sheath diameter was between 19 and 24 F [30]. They concluded that the only independent predictive parameters for minor and major vascular complications in their study population of 375 subjects were sheath diameter ≥ 19 F and SFAR ≥ 1.05. In line with these results, Sari et al (2015) observed a complication rate of 10.1% in their entire study population of 127 patients, and their results demonstrated that both the sheath size and the SFAR were predictive factors. According to our multivariate analysis there was no predictive parameter. In our opinion, this can be explained by the reduction of the vascular complications when using the Proglide^®^ system and by respecting the anatomical limits of the vessels. These conflicting results imply that more large-scale comparative studies are necessary to clearly define the impact of the sheath diameter as such and/or the SFAR on the prevalence of vascular complications during TAVI.

The new devices by Edwards and Medtronic use sheaths with smaller diameters (14–16 F and 14 F, respectively). This minimization of sheath diameter is expected to result in a further reduction of vascular complications. However, in a study by Kodali et al., the rate of major vascular complications using the 14 and 16 F sheath with the Sapien 3 valve was 6.1% [37] and in the study of Manoharan et al. using the 14F Medtronic Evolute R valve it was 8.3% [38]. These rates are higher compared to 3.8% as determined in our study. However, in the other studies, the closure device system, which significantly influences the rate of vascular complications [31], is not mentioned. Further studies comparing the different sheath devices and diameters are needed to analyze the extent of these influencing parameters on vascular complications.

### Study limitations

This study has some limitations. First, it is a retrospective, non-randomized study, and therefore selection bias cannot be excluded. A randomized prospective study or propensity score matching would provide a more reliable analysis. Second, our study was performed as a single-center analysis and includes a limited study population. Large-scale studies are required to confirm our results.

### Conclusions

In the present study including 183 patients, the sheath diameter (18 F vs. 19–24 F) had no impact on the incidence of minor or major vascular complications during TF-TAVI using the Perclose-Proglide^®^ closure device. Not even 30-day and long-term mortality were influenced by the sheath diameter. Further studies with even smaller calibers of delivery systems down to 14 F are required to reevaluate these results.
